# Late-onset Hailey-Hailey disease with a novel ATP2C1 mutation in an older female patient

**DOI:** 10.1016/j.jdcr.2023.11.036

**Published:** 2024-01-23

**Authors:** Tomohisa Horikawa, Sayaka Yamaguchi, Takuya Omine, Takuya Miyagi, Daisuke Utsumi, Kenzo Takahashi

**Affiliations:** Department of Dermatology, Graduate School of Medicine, University of the Ryukyus, Okinawa, Japan

**Keywords:** ATP2C1, autosomal dominant disease, familial benign chronic pemphigus, Hailey-Hailey disease

## Introduction

Hailey-Hailey disease (HHD), also termed familial benign chronic pemphigus, is a rare autosomal, dominant, heritable, erosive skin disorder. It is caused by mutations of the ATPase calcium-transporting type 2C member 1 (ATP2C1) gene, which encodes the Ca^2+^/Mn^2+^-ATPase 1 pump expressed in the Golgi apparatus of keratinocytes. Thus far, 180 pathogenic mutations have been reported in this gene.^1^ In this study, we identified a novel genetic mutation in a case of HHD that manifested in an elderly female patient. We further endeavored to analyze the reported mutation from a 3-dimensional structural perspective.

## Case report

**Fig 1:**
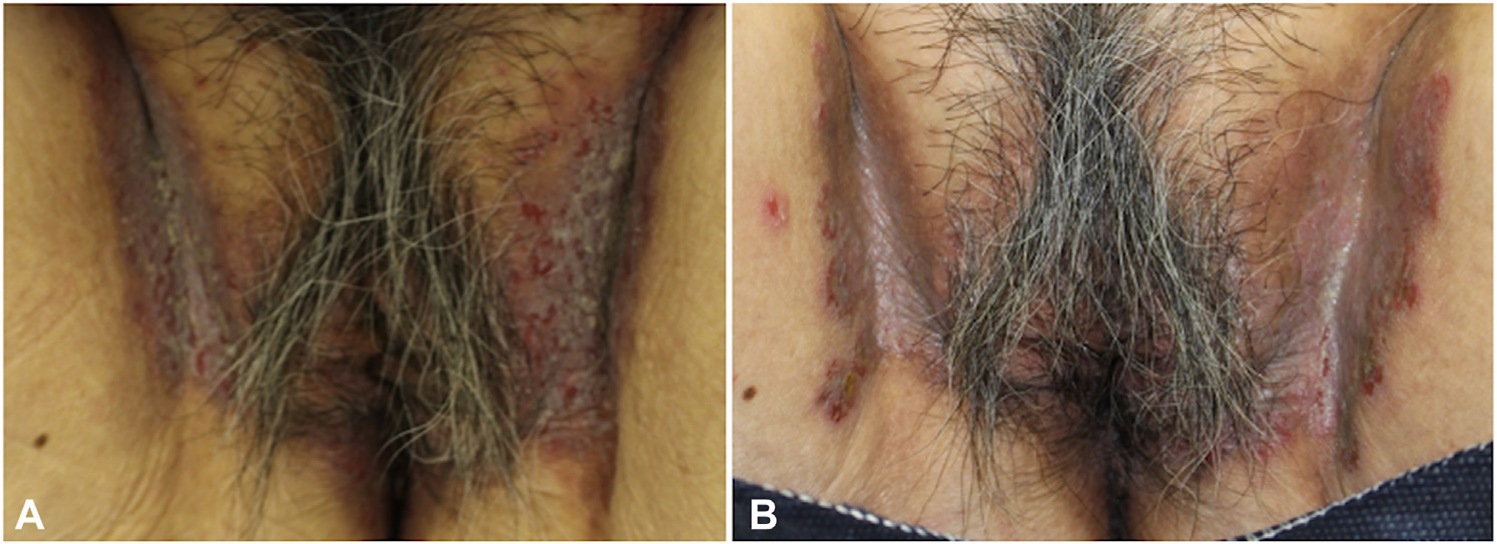
Clinical manifestations. **A**, Erythematous erosive plaques with fissures on the bilateral groin at the initial visit. **B**, Persistence of erosion and cracks following topical antifungal therapy.

**Fig 2:**
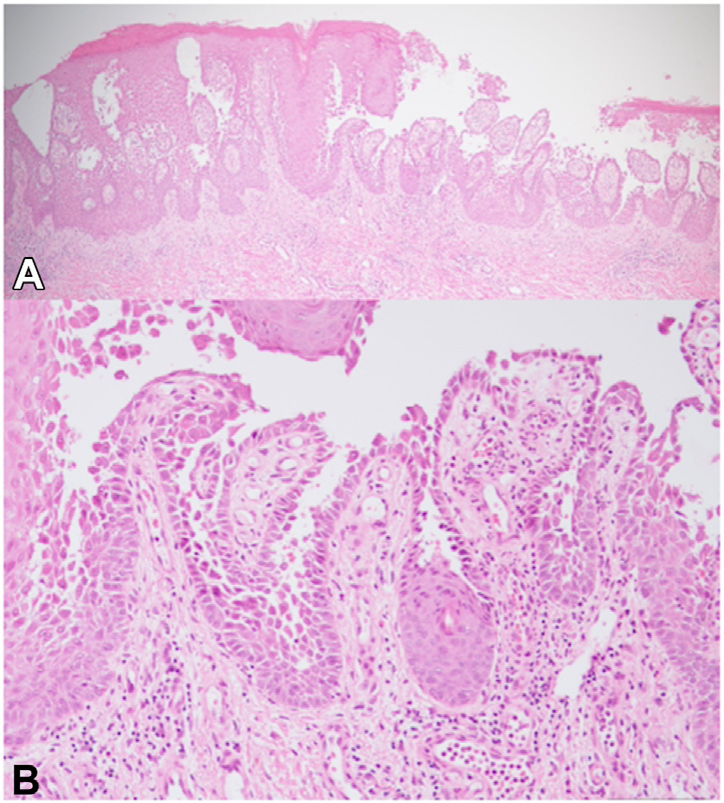
**A**, Histopathologic examination revealed the presence of acantholysis, where 1 or 2 layers of basal cells remained (hematoxylin and eosin, ×100). **B**, The dermal papilla was villous (hematoxylin and eosin, ×200).

A 70-year-old Japanese female presented with a 6-month history of erythematous rash on her groin. Treatments with a topical corticosteroid and an antibiotic were ineffective, so the patient was referred to our hospital. Physical examination revealed erythematous, erosive plaques with fissures on the bilateral groin (Fig 1, *A*). There were no other lesions on the axilla or inframammary regions. The patient had been otherwise healthy and her siblings exhibited no such symptoms. The KOH examination yielded a positive result for Candida and the patient underwent 1 month of topical antifungal therapy. As erosion and cracking persisted on the margin lesion (Fig 1, *B*), a skin biopsy was conducted. The histopathological examination revealed the presence of suprabasal clefts, acanthosis and hyperkeratosis, infiltration of lymphocytes, neutrophils, and eosinophils in the superficial dermal layer, with no deposits of immunoglobulins or complements detectable via direct immunofluorescence examination (Fig 2, *A* and *B*).

DNA was extracted from peripheral blood lymphocytes and a whole exome sequence analysis was performed. A heterozygous single-nucleotide substitution of c.2207 T > C was found in exon 23 of the ATP2C1 gene, which converts phenylalanine to serine (p.F736S) in the secretory pathway Ca^2+^/Mn^2+^-ATPase pump type 1 (SPCA1). This mutation was previously unknown. Eventually, the patient was diagnosed with HHD. After the Candida infection subsided, the patient applied topical steroids and cooling therapy to the groin, leading to the disappearance of symptoms. Subsequently, the patient experienced minimal symptoms in her groin area, with occasional erythema appearing in the axilla. The condition was alleviated through the application of topical steroids.

## Discussion

More than 80% of HHD cases develop before the age of 50.^2^ The latest reported onset of HHD was at 78 years of age^3^; unfortunately, no detailed investigation of genetic mutations was conducted in that patient. The present case had a novel mutation and very late onset of the disease.

Missense mutation in ATP2C1 are frequently located in exons 12, 13, 18, 21, and 23.^4^ It is believed that missense mutations in these regions significantly affect the function of SPCA1. There have been 12 reported mutations in exon 23 of ATP2C1, including our own case.^4^ These mutations comprise 7 missense mutations, 4 premature termination codons resulting from nonsense mutations, insertions, or deletions, and 1 amino acid deletion. Five of the cases with missense mutations in exon 23 developed in their 20s and 30s, and no common trends were observed with our own cases of later onset.

We compared the nature of genetic mutations in all age groups^4^ and in patients with an onset age of 50 years or older. Among all age groups, 49 (29.5%) out of 166 genetic mutations were missense mutations, and the same tendency was observed in 6 out of 19 (31.6%) patients aged 50 years or older at onset, with no specific characteristics found in the older age group at onset.

**Fig 3:**
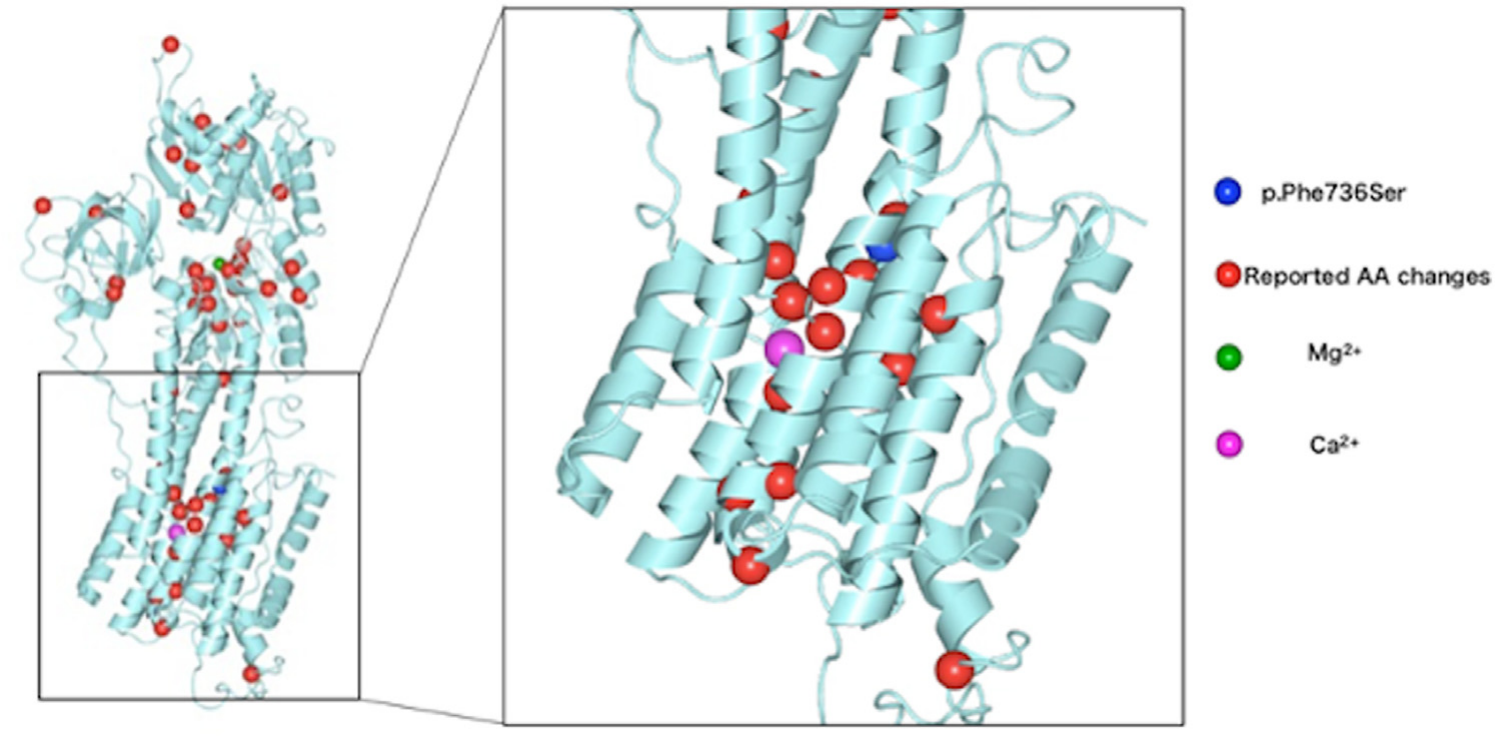
Predicted 3-dimensional structure of the SPCA1 protein. The known missense mutations of Hailey-Hailey disease are plotted on the SPCA1 protein structure. In our case, the mutation (p.F736S) was located in the transmembrane domain and in the vicinity of the selective uptake of calcium ions.

As we consequently suspected that the observed structural change in the mutated SPCA1 protein might be somewhat unique and exert a subtle effect, we investigated the 3-dimensional (3D) structure of the protein. However, because there was no registered 3D structure of SPCA1 in the Protein Data Bank, which is the basis for the 3D structure analysis software Waals (Altif Labs, Inc), we used the structure of human SERCA2 (Darier disease-causing protein), which shares the highest homology with SPCA1, for the 3D structure model.^5^ The previously reported missense mutations causing HHD^6^ and our case were placed on the SERCA2 structure (Fig 3). The mutation (p.F736S in SPCA1) in our case was located in the fifth transmembrane domain in the vicinity of the residue responsible for the selective uptake of calcium ions. Numerous known missense mutations that cause the typical and more severe HHD phenotypes were also located in regions neighboring this residue. Therefore, we concluded that this mutation may not necessarily evoke a weak structural change. We also performed 3D structural analyses of older-onset (R143P)^7^ and younger-onset (G220 E)^1^ cases and found no significant structural differences in either. Further protein structural and functional analyses may make it possible to determine whether the late onset of HHD in this case was due to a structural change caused by this mutation. Alternatively, other factors may have led to the late onset of the disease, such as the transcriptional activity of the wild-type ATP2C1 allele or the presence of cellular machinery other than SPCA1 that regulate intracellular calcium.

## Conflicts of interest

None disclosed.
